# Assessing Changes in Characteristics of Hot Extremes Over India in a Warming Environment and their Driving Mechanisms

**DOI:** 10.1038/s41598-020-59427-z

**Published:** 2020-02-14

**Authors:** Manish K. Joshi, Archana Rai, Ashwini Kulkarni, Fred Kucharski

**Affiliations:** 1 Indian Institute of Tropical Meteorology, Pune, Maharashtra India; 2 Earth System Physics Section, Abdus Salam International Centre for Theoretical Physics, Trieste, Italy; 3 Center of Excellence for Climate Change Research/Department of Meteorology, King Abdulaziz University, Jeddah, Saudi Arabia

**Keywords:** Environmental sciences, Climate sciences, Atmospheric science, Climate change

## Abstract

Change in hot extremes is one of the accepted evidence and also a global indicator of an anthropogenic climate change, which has serious environmental and economic impacts. In the present study, the India Meteorological Department gridded temperature data is used to characterize hot extremes over India in terms of frequency and intensity. Results provide compelling evidence that large parts of India, except the Indo-Gangetic plains, have experienced more occurrences of hot days (upsurge by 24.7%) having higher temperatures in the recent period (1976–2018), compared to the past (1951–1975), which suggests a shift in climate. Strong positive geopotential height anomalies at 500 hPa over the northern parts of India, which dynamically produces subsidence and clear sky conditions along with reduced precipitable water and depleted soil moisture are identified to be the crucial factors responsible for an increase of hot extremes in recent decades. Furthermore, the preceding December-February Niño-3.4 sea surface temperature (SST) anomalies are strongly connected with hot days frequency and the mechanism for the lag of several months is related to 3–4 months delayed response of Indian Ocean SSTs to El Niño/Southern Oscillation. Thus, post-Niño hot extremes over India can be potentially anticipated in advance and this will help society to prepare for such extremes.

## Introduction

According to the fifth assessment report of the Intergovernmental Panel on Climate Change (IPCC), the globally averaged combined land and ocean surface temperature data exhibits warming of 0.85 °C during 1880–2012^1^. In a warming environment, the temperature extremes are anticipated to change in terms of severity, frequency, and duration probably due to the shift in probability distribution towards higher values, which will likely cause the augmentation and reduction of hot and cold events, respectively^2–5^. Extreme heat events such as those observed in Western Europe in 2003^6–8^ and Russia in 2010 caused the death of thousands of human beings as well as crop failure and showed how destructive impact it would leave on our societies^9^. Previous studies reported a rapid increase in hot extremes almost all over the globe^2,3,10,11^. Even during the most debatable global warming hiatus, the hot extremes continuously increased unabatedly over land^12^. However, at regional scales, the change in temperature extremes affects society more explicitly than does the mean climate change. Recent studies suggest that hot extremes have increased over many regions like South Asia^13^, China^14^, South Korea^15^, the United States^16^, etc.

India is also experiencing the warming climate with an increase in the annual mean, minimum, and maximum temperatures from the beginning of the 20^th^ century and in recent decades^17–19^. Long observational records reveal a significant increasing trend in the mean (0.48 °C), minimum (0.22 °C), and maximum (0.74 °C) temperatures over India during 1901–2003^18^. In recent decades, India has been considerably affected by hot extremes that killed more than thousands of people in the years 1998, 2010, 2013, and 2015^20–22^. The station data over India shows an increasing trend in the frequency of hot days (especially over the eastern and western coasts as well as over the interior peninsula)^23^ and a widespread increase in the intensity of those events (i.e., out of 121 stations considered, 70% of stations illustrate an increasing trend) during the last three decades of the 20^th^ century^24^. Later on, after the availability of India Meteorological Department (IMD) gridded temperature data, a study reported a significant increasing trend in the number of hot extremes only in the interior peninsula during 1969–2005; however, the entire country experienced the maximum number of hot days during 1996–2005^25^. A recent study attributed the upsurge in the concurrent hot day and night events over India to anthropogenic emissions and stated that these hot extremes are going to increase at a much faster rate under global warming scenarios^21^. A heat wave is a particular condition of extreme temperature events, associated with hot sustained temperatures for a prolonged period and this precise condition is also observed over many parts of the globe^3,26^, including India^20,27^.

Literature suggests that one of the most crucial factors responsible for an extreme temperature event is the large-scale circulation of the atmosphere^14,15,28^. Climate models also elucidate an increase in extreme temperature events in future scenarios^21,24^, but the changes are not uniformly distributed and will be affected by alterations in the large-scale atmospheric circulation^3^. The temperature extremes over China^14^ and South Korea^15^ are also linked with variations in the occurrence of atmospheric patterns. Besides this, natural variability like El Niño/Southern Oscillation (ENSO) also plays a crucial role in modulating temperature extremes worldwide^29–31^ as well as over India^32^.

So far the hot extremes over India are generally scrutinized from a statistical perspective and mainly for the last three decades of the 20^th^ century. Besides this, previous studies^20,23–25^ hardly provide the underlying mechanisms responsible for such hot extremes. Thus, to better anticipate hot extremes a deeper study is required at regional scales to comprehend the physical mechanisms allied with them. This study supersedes previous studies because here an attempt has been made to diagnose the change in the characteristics of hot extremes over India before and after the 1976 climate shift. Additionally, special emphasis is given to understand the driving mechanisms responsible for such changes. Thus, in this perspective, the present study addresses the following questions: (1) Does the probability distribution of daytime maximum temperatures over India for the April-June (AMJ) season has shifted towards higher values in the recent climate, compared to the past? (2) If yes, then what is the effect of this shift on the characteristics (i.e., frequency and intensity) of hot extremes? (3) What are the driving mechanisms (including atmospheric circulations, local factors, and natural variability) responsible for the change in hot extremes over India?

## Results and Discussion

### Probability distribution of daytime maximum temperatures

**Figure 1:**
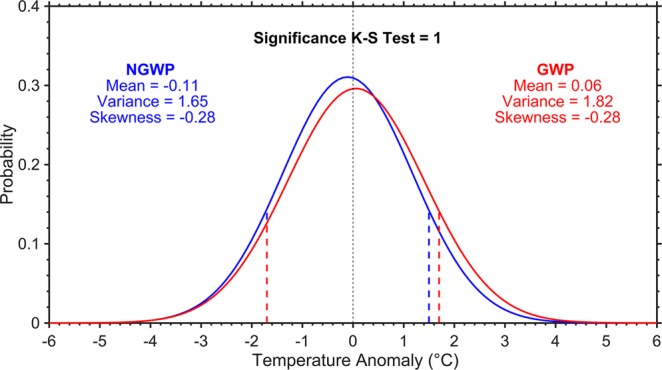
Shift in the probability distribution of daytime maximum temperatures. Probability density functions of daily maximum temperature anomalies during the non-global warming period (NGWP: 1951–1975; blue) and global warming period (GWP: 1976–2018; red) for the AMJ season. The K-S = 1 reveals that the probability density functions for the two periods are statistically different from each other at the 95% confidence level. The vertical lines represent the 10^th^ and 90^th^ percentiles of the respective distribution.

The Probability Density Functions (PDFs) of daily maximum temperature anomalies during the non-global and global warming periods (see Methods) for the AMJ season, along with the three-moment statistics related to the location (i.e., mean), scale (i.e., variance), and shape (i.e., skewness) parameters are shown in Fig. 1. Figure 1 highlights a positive shift in the location of the PDF (i.e., an increase in the mean daily maximum temperature anomaly by 0.17 **°**C), which has led to new records of higher temperatures in the recent period, compared to the past. Along with mean, the variance has also increased (i.e., the PDF has become wider), which affects the probability of extreme events, with more frequent hot days having more extreme high temperatures and lesser cold days in the AMJ season. During both periods, the distribution is negatively skewed, but the asymmetry around the respective means appears to be the same indicating that there is no change in the shape of PDFs. Generally, the alteration in location, scale, or shape of the distribution has a larger impact on temperature extremes than on the mean. Thus, to determine whether the distributions of the two periods are statistically different from each other at the 95% confidence level, a non-parametric two-sample K-S test is applied (see Methods). The K-S test reveals that the temperatures are significantly different in the recent period as compared to the past.

In the global warming period, the upper (90^th^) and lower (10^th^) threshold percentiles are increased by 0.22 and 0.05 **°**C, respectively. The extreme temperatures have increased in such a way that the 90^th^ (10^th^) percentile of the non-global warming period is the 86^th^ (9.4^th^) percentile of the global warming period. In other words, 14% (9.4%) of values during the global warming period lie above (below) the 90^th^ (10^th^) percentile of the non-global warming period.

As per our knowledge, none of the previous studies have shown the actual shift in the daily maximum temperature distribution over India. However, using a global dataset (HadGHCND) of daily maximum temperature anomalies it was reported that the temperature distribution has significantly shifted towards higher values in the recent climate, compared to the past, over the globe, tropics, and the northern/southern hemisphere extratropics, nonetheless, the HadGHCND dataset has insufficient coverage over most parts of India^33^.

### Changes in the characteristics of hot extremes

Since there is a significant shift in the probability distribution of daytime maximum temperatures in recent decades, therefore, the change in the characteristics of hot extremes (especially on their occurrences, corresponding intensities, and on the seasonal maximum intensity) in the global warming period compared to non-global are explored in this section.

**Figure 2:**
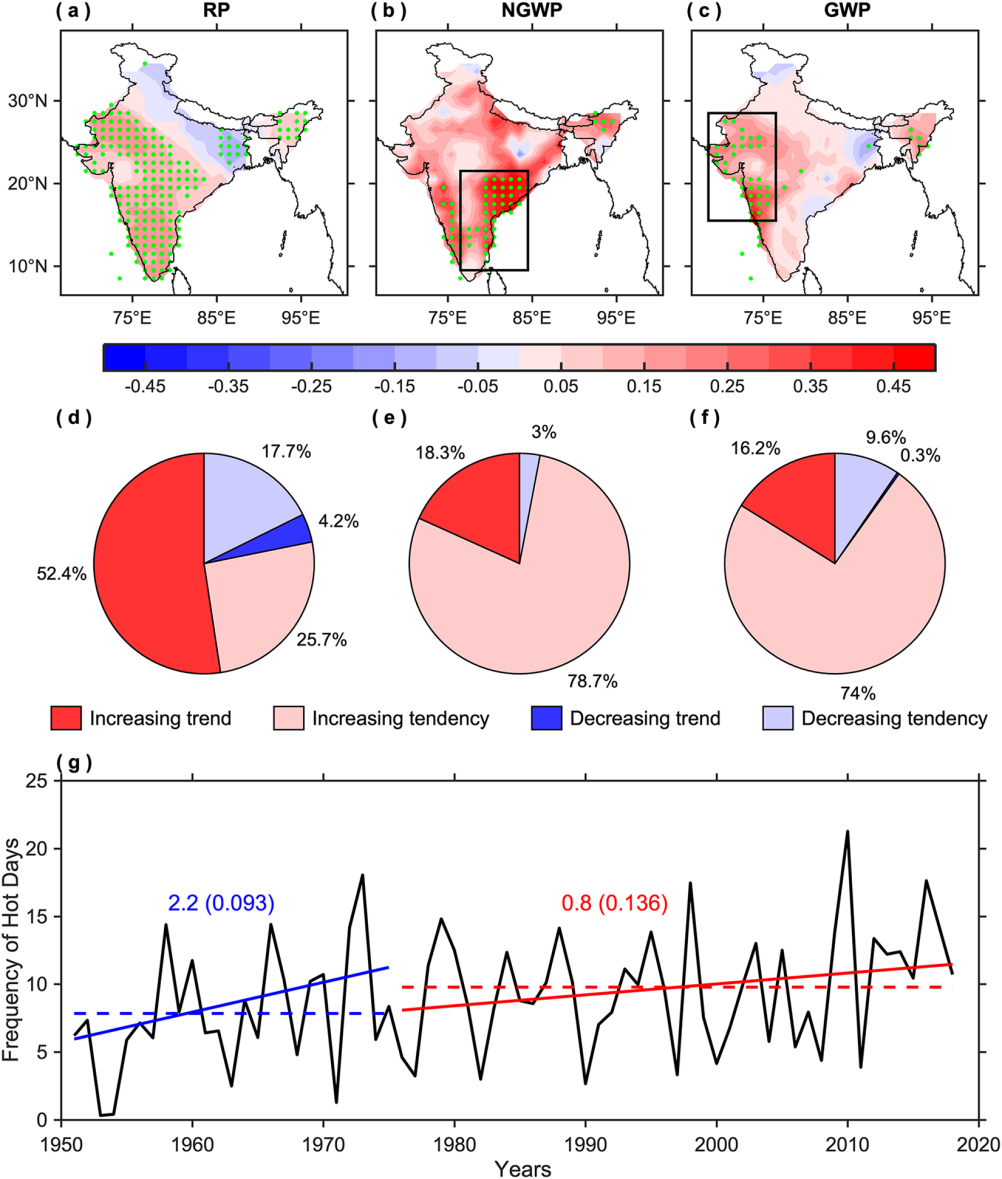
Spatiotemporal trends in the frequency of hot days. (**a**–**c)** Spatial patterns of temporal trends (number of hot days per year) and (**d**–**f**) corresponding pie charts, illustrating the number of grids (in percent) having increasing and decreasing trend/tendency, in the frequency of hot days for the AMJ season during the reference period (RP: 1951–2018), non-global warming period (NGWP: 1951–1975), and global warming period (GWP: 1976–2018). The green stippling indicates the grid points where the trends are statistically significant at the 95% confidence level. (**g**) Variation (black) and linear trends in the frequency of hot days, area-averaged over all-India, during the non-global (blue) and global (red) warming periods. Values along the trend lines indicate the amount of change in the number of hot days per decade and those in parentheses represent its p-values. The dashed lines indicate the average frequency of hot days in the non-global (blue) and global (red) warming periods. This figure was prepared using the MATLAB version R2017a software (http://in.mathworks.com).

The spatial patterns of temporal trends in the frequency of hot days for the AMJ season during the reference, non-global, and global warming periods are illustrated in Fig. 2a–c. During the reference period, a widespread significant increase in the frequency of hot days is observed over all-India, except over the Indo-Gangetic plains. On the other hand, during the non-global warming period, only the eastern and southern parts of India (i.e., over interior peninsula, west coast, and east coast) have experienced significant increase in the frequency of hot days; whereas in the global warming period, hot days have significantly increased over the northwestern parts of Interior Peninsula and along the western coast, indicating a spatial shift of significant increasing trends. Consistent with all-India, the probability distributions for southeast (9.5°N–21.5°N, 76.5°E–84.5°E; box shown in Fig. 2b) and western (15.5°N–28.5°N, 68.5°E–76.5°E; box shown in Fig. 2c) India also reveal a positive shift in the location of PDF and an increase in variance (Fig. S1a,b), implying more frequent hot days having higher temperatures in recent decades over the respective regions. Though the spatial pattern (Fig. 2b,c) and temporal variation (Fig. S2a,b) illustrate that the hot days frequency has significantly increased over southeast and western India during the non-global and global warming periods, respectively; the average frequency of hot days for both the regions (i.e., southeast and western India) is comparatively larger in recent decades (10.76 and 10.51 hot days), compared to the past (6.15 and 6.58 hot days).

During the reference period, 52.4% (25.7%) of grids show an increasing trend (tendency) in the occurrence of hot days (Fig. 2d), which is 34.1% and 36.2% (53% and 48.3%) more (less) as compared to the non-global (Fig. 2e) and global warming (Fig. 2f) periods, respectively. This implies that there is a marginal decrease of 2.1% (4.7%) in the number of grids showing an increasing trend (tendency) in the frequency of hot days during the global warming period, compared to non-global.

Figure 2g divulges the variation and linear trend in the frequency of hot days, area-averaged over all-India. In the non-global warming period, hot days have significantly increased at a much faster rate (2.2 hot days per decade), compared to the global warming period (0.8 hot days per decade), but the average frequency of hot days in the recent period (9.78 hot days; shown by red dashed line) is significantly more (fails to cross the 95% confidence level, but significant at 90%, using Student’s t-test for unequal variance) as compared to the past (7.84 hot days; shown by blue dashed line).

Figure S3 shows the spatial patterns of temporal trends in the average intensity of hot days for the AMJ season during the reference, non-global, and global warming periods. Average intensity is defined as the seasonal mean of maximum temperature, corresponding to grids that exceed the 90^th^ percentile threshold of maximum temperature for the reference period. During the reference period as well as in the non-global and global warming periods, the intensity of hot days (Fig. S3a–c) has generally increased over the regions where its occurrence has increased, except over the Indo-Gangetic plains and along the western coast in the non-global warming period. Concisely, the number of grids divulging an increasing trend/tendency in the average intensity of hot days during the global warming period (Fig. S3f) has substantially increased by 22.8% and 9% as compared to non-global warming (Fig. S3e) and reference (Fig. S3d) periods, respectively.

**Figure 3:**
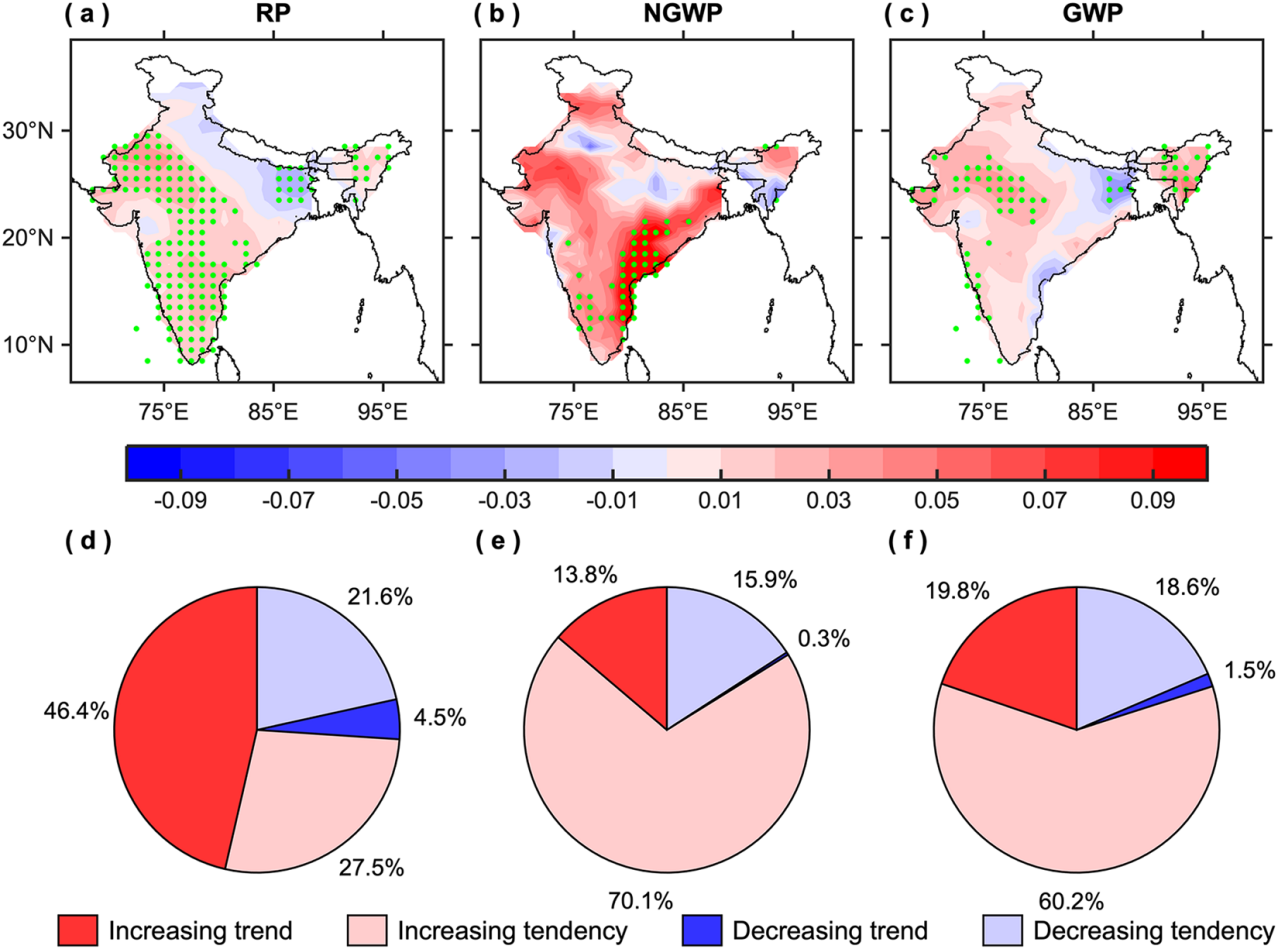
Trends in the seasonal maximum intensity. **(a**–**c)** Spatial patterns of temporal trends (°C/yr) and (**d**–**f**) corresponding pie charts, illustrating the number of grids (in percent) having increasing and decreasing trend/tendency, in the maximum intensity for the AMJ season during the reference period (RP: 1951–2018), non-global warming period (NGWP: 1951–1975), and global warming period (GWP: 1976–2018). The green stippling indicates the grid points where the trends are statistically significant at the 95% confidence level. This figure was prepared using the MATLAB version R2017a software (http://in.mathworks.com).

Likewise the trend in the frequency of hot days, the trends in the seasonal maximum intensity are also homogeneous in sign over most parts of India during the three periods (Fig. 3a–c), except over the Indo-Gangetic plains in the reference period and over its western and eastern sides during the non-global and global warming periods, respectively. In contrast to the average intensity of hot days, significant trends in the seasonal maximum intensity are generally observed over the regions where the frequency of hot days has increased significantly. On the whole, 73.9% of grids show an increasing trend/tendency in the seasonal maximum intensity during the reference period (Fig. 3d), which is 10% and 6.1% less as compared to the non-global and global warming periods (Fig. 3e,f), respectively. In contrast to the average intensity of hot days, the number of grids having an increasing trend/tendency in the seasonal maximum intensity has marginally decreased by 3.9% in the global warming period, consistent with the frequency of hot days.

Further, the spatial consistency between the frequency of hot days and their corresponding average intensity as well as the seasonal maximum intensity for all periods considered is assessed using cross-tabulation and chi-square statistics against the null hypothesis (H_0_), which states that the table is independent in each dimension (for details see Tables S1 and S2). Besides this, the spatial consistency between the non-global and global warming periods for the frequency of hot days, their average intensity, and seasonal maximum intensity are also evaluated and tabulated in Table S3.

**Figure 4:**
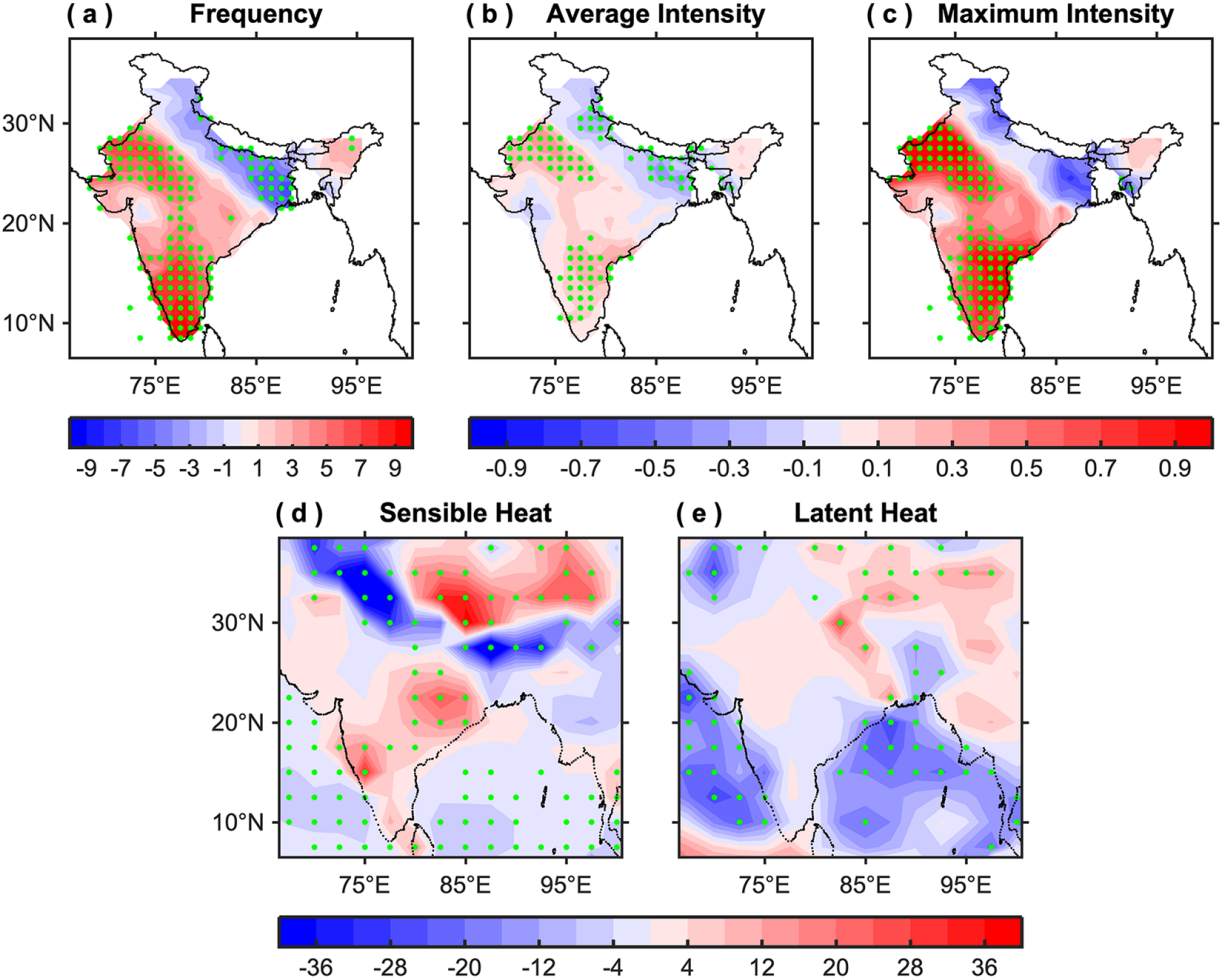
Changes in the characteristics of hot extremes and associated heat fluxes. Climatological mean difference between the global and non-global warming periods of (**a**) frequency of hot days, (**b**) average intensity of hot days (°C), (**c**) maximum intensity (°C), (**d**) sensible heat flux (W/m^2^), and (**e**) latent heat flux (W/m^2^) corresponding to the hot days for the AMJ season. The green stippling indicates the grid points where the difference is statistically significant at the 95% confidence level. This figure was prepared using the MATLAB version R2017a software (http://in.mathworks.com).

Moreover, the climatological mean difference between the global and non-global warming periods in the frequency of hot days (Fig. 4a), the average intensity of hot days (Fig. 4b), and the seasonal maximum intensity (Fig. 4c) reveals that though the frequency/intensity of hot days have increased over most parts of India at a much faster rate during the non-global warming period, but their average during the global warming period is significantly higher, except over the Indo-Gangetic plains. This infers a clear shift in the climate towards warmer temperatures, i.e., most parts of India (except the Indo-Gangetic plains) have experienced more occurrences of hot days having higher temperatures in the recent climate. In other words, India has become more prone to warmer temperatures. The probable reason why the frequency/intensity of hot days over the Indo-Gangetic plains is significantly less during the global warming period, compared to non-global, will be discussed in detail in the next paragraph.

The Indo-Gangetic plain is amongst the most heavily irrigated regions in the world^34,35^. Irrigation affects the surface energy budget by enhancing latent heat flux and deteriorating sensible heat flux^36^. Due to intensive irrigation over the Indo-Gangetic plains, the vegetation and evapotranspiration have increased substantially^37–39^. This increase in evapotranspiration has led to the smaller portion of sensible heat flux (Fig. 4d) versus latent heat (Fig. 4e) over most parts of the Indo-Gangetic plains during the global warming period as compared to non-global (for details see Fig. S4), which has resulted a decrease in near-surface air temperatures over that region.

### Physical mechanism associated with hot extremes

Generally, the hot extremes are studied from the climatological point of view, but fundamentally they are meteorological events^26^. Therefore, to understand the physical mechanism associated with hot extremes over India, the crucial atmospheric circulation patterns are scrutinized and compared in the reference, non-global, and global warming periods.

**Figure 5:**
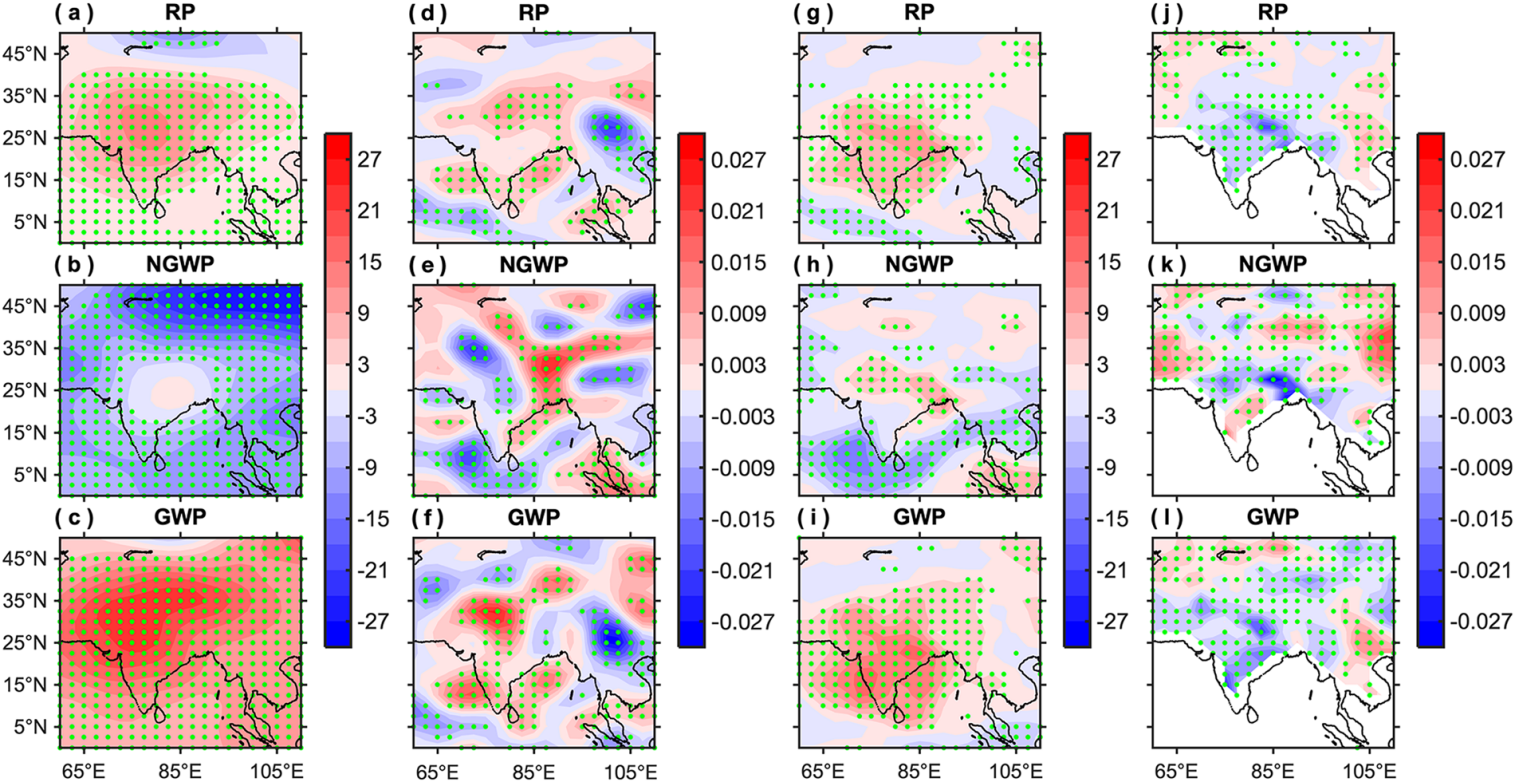
Composites of atmospheric and surface parameters allied with hot extremes. Composites of (**a**–**c**) geopotential height (m) anomalies at 500 hPa, (**d**–**f**) vertical velocity (Pa/s) anomalies at 500 hPa, (**g**–**i**) upward longwave radiation flux (W/m^2^) anomalies at top of the atmosphere, and (**j**–**l**) soil moisture (fraction; 0–10 cm) anomalies corresponding to hot days for the AMJ season during the reference period (RP: 1951–2018), non-global warming period (NGWP: 1951–1975), and global warming period (GWP: 1976–2018), respectively. The green stippling indicates the grid points where the composite is statistically significant at the 95% confidence level. This figure was prepared using the MATLAB version R2017a software (http://in.mathworks.com).

In contrast to non-global, global warming and reference period’s composites show strong positive geopotential height anomalies (Fig. 5a–c) over the northern parts of India at 500 hPa. This amplification of the positive 500 hPa height anomalies in the global warming period (Fig. 5c) is associated with higher than normal frequency (Fig. 4a)/intensity (Fig. 4b,c) of hot days over India for the recent compared to past climate. In general, the anomalous high pressure over the northern parts of India is allied with sub-tropical high, which is usually referred to as persistent high^26,40^. This persistent high-pressure system, linked with the anti-cyclonic flow, in the middle troposphere is a key synoptic component for the extreme hot events, which causes descending motion that leads to surface warming due to adiabatic compression.

To verify this, the composites of vertical velocity anomalies at 500 hPa are computed as shown in Fig. 5d–f. In contrast to non-global, global warming and reference periods divulge positive omega values over the Indian landmass that indicates sinking motion, i.e., subsidence over the respective region. Furthermore, this descending motion is allied with clear skies, which is well depicted in the upward longwave radiation flux anomalies (Fig. 5g–i). The strong positive upward longwave radiation flux anomalies, in the recent period (Fig. 5i), indicate the presence of comparatively less clouds as compared to the past (Fig. 5h). As a consequence, more solar radiation energy has been received at the Earth’s surface during recent decades (Fig. S5a–c), which has led to warmer temperatures over the Indian landmass (in agreement with Fig. 1). This is also quite consistent with Fig. 4a–c, revealing that India has experienced more frequent hot days having higher temperatures in the recent climate.

Besides this, the composites of soil moisture are also computed for the respective periods (Fig. 5j–l). Compared to the past (Fig. 5k), the reference period (Fig. 5j) as well as the recent climate (Fig. 5l) reveals depleted soil moisture. In general, soil moisture controls the amount of sensible and latent heat fluxes into the atmosphere. Thus, the depleted soil moisture in the recent period has led to the larger proportion of sensible heat flux compared to latent over most parts of India (except the Indo-Gangetic plains; Fig. 4d,e), which induces positive feedback between the atmospheric heating and further drying of the soil. As a consequence, the near-surface air temperatures have increased in recent decades (consistent with Fig. 1; divulging a positive shift in the location of the PDF), which in turn provides a direct association between the soil moisture and near surface-air temperatures. Though the soil moisture coupling is ostensible through the entire range of temperature, it is particularly more pertinent for extremely hot temperatures^41–45^. Thus, the amalgamation of persistent high-pressure along with low soil moisture conditions intensifies the positive feedback and augments the surface warming and hence plays an important role in the frequency and intensity of hot extremes.

**Figure 6:**
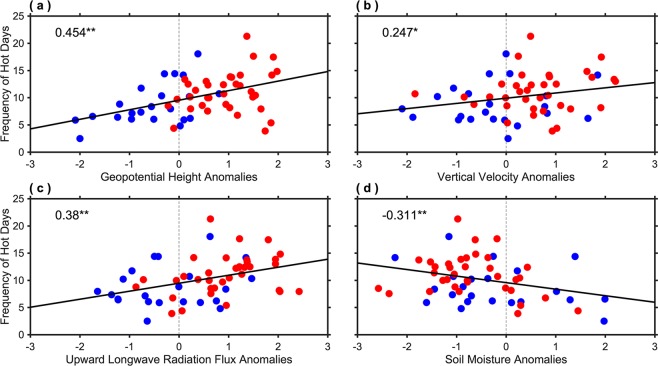
Relationship of atmospheric and surface parameters with hot extremes. Scatter plot of the frequency of hot days, area-averaged over all-India (shown in Fig. 2g) versus the standardized mean of (**a**) geopotential height (m) anomalies at 500 hPa, area-averaged over 10°N–32.5°N and 65°E–100°E; (**b**) vertical velocity (Pa/s) anomalies at 500 hPa, area-averaged over 10°N–32.5°N and 70°E–85°E; (**c**) upward longwave radiation flux (W/m^2^) anomalies, area-averaged over 10°N–32.5°N and 65°E–100°E; and (**d**) soil moisture (fraction) anomalies, area-averaged over 10°N–32.5°N and 70°E–85°E corresponding to the hot days for the AMJ season. The correlation values shown by * (**) are statistically significant at the 90% (95%) confidence level. The blue and red scatters represent the non-global and global warming years, respectively.

Further, to investigate the relationship of these atmospheric and surface variables with hot extremes several scatter plots have been considered, for example, the frequency of hot days, area-averaged over all-India (Fig. 2g) versus the mean of (a) geopotential height anomalies at 500 hPa, area-averaged over 10°N–32.5°N and 65°E–100°E; (b) vertical velocity anomalies at 500 hPa, area-averaged over 10°N–32.5°N and 70°E–85°E; (c) upward longwave radiation flux anomalies, area-averaged over 10°N–32.5°N and 65°E–100°E; and (d) soil moisture anomalies, area-averaged over 10°N–32.5°N and 70°E–85°E corresponding to hot days for the AMJ season (Fig. 6a–d). For the first three scatter plots, the relationship is positive having correlations 0.454 (significant at the 95% confidence level), 0.247 (fails to cross the 95% confidence level, but significant at 90%), and 0.38 (significant at the 95% confidence level), respectively. This exemplifies that, in general, larger geopotential height, vertical velocity, and upward longwave radiation flux will produce a larger number of hot days over India. On close inspection, it can be seen that during most of the non-global warming years these variables have smaller values, compared to global warming years, which is quite consistent with the composites shown in Fig. 5. This is also in agreement with Figs. 2g and 4a–c revealing why the non-global warming period has experienced a significantly less average number of hot days compared to the global warming period. On the other hand, the soil moisture is anti-correlated with the frequency of hot days having a correlation of −0.311 (significant at the 95% confidence level). This signifies that during the years when the soil is wet (or having higher values), India has experienced less frequency of hot days. As discussed earlier, the augmented (depleted) soil moisture will lead to a larger proportion of latent (sensible) heat flux, compared to sensible (latent)^43^. This is because, the soil moisture provides moisture for evaporation due to which some portion of the incoming solar radiation energy reaching the Earth’s surface is utilized in evaporating the water in the soil instead of heating the ground and subsequently the near-surface air is warming less than over a dry surface, which limits the maximum temperatures that in turn limits the hot extremes.

Humans are very sensitive to humidity because their ability to cool themselves by sweating (evaporative cooling) greatly depends on it. In particular, relative humidity is an important stress factor for thermal regulation and can influence human health because it directly affects our thermal comfort. Hence as compared to hot and dry conditions, equivalently hot and humid conditions can be more dangerous.

Therefore, the composite of precipitable water vapor (Fig. S5d–f) and relative humidity (Fig. S5g–i) have been constructed for the respective periods. Compared to the past climate, recent and the reference periods depict reduced precipitable water anomalies over the Indian landmass. On the other hand, the recent climate over India has become significantly warmer as compared to the past (Fig. 4b,c). This implies that the saturated vapor pressure (depends on temperature and it increases with its increase) is larger in recent decades. Henceforth, the lower (higher) precipitable water values over the Indian landmass along with the higher (lower) temperatures have contributed to lower (higher) relative humidity values in the recent (past) period (Fig. S5g–i). Thus, the reduced humidity in the recent period may offset some of the effects of augmented temperature by permitting more evaporation and thereby enhancing the effectiveness of the mechanism associated with thermoregulation.

Though in some regions a positive relation between precipitable water vapor and air temperature has been identified, particularly in the arid regions^46^, our findings presented in Figs. 5 and S5 clearly indicate that for the AMJ season hot day events are characterized by sinking motion and reduced moisture as well as clouds. To develop this argument even further, the composites of surface downward longwave radiation have been computed, which should provide warming if a water vapor greenhouse effect is present (Fig. S5j–l). However, the downward longwave radiation forcing provides a cooling tendency during the reference and global warming periods (Fig. S5j,l), which is consistent with the reduced water vapor in the atmosphere (Fig. S5d,f). On the other hand, the composites of surface downward solar radiation (Fig. S5a–c) indicate strong warming due to this mechanism, and this is even enhanced in the global warming period (Fig. S5c). Therefore, even though our composite analysis does not strictly provide cause-and-effect relationships, it is physically sound to assume that the increased sinking motion-induced cloud cover reduction and therefore solar radiation increase are the main mechanisms for the hot days.

**Figure 7:**
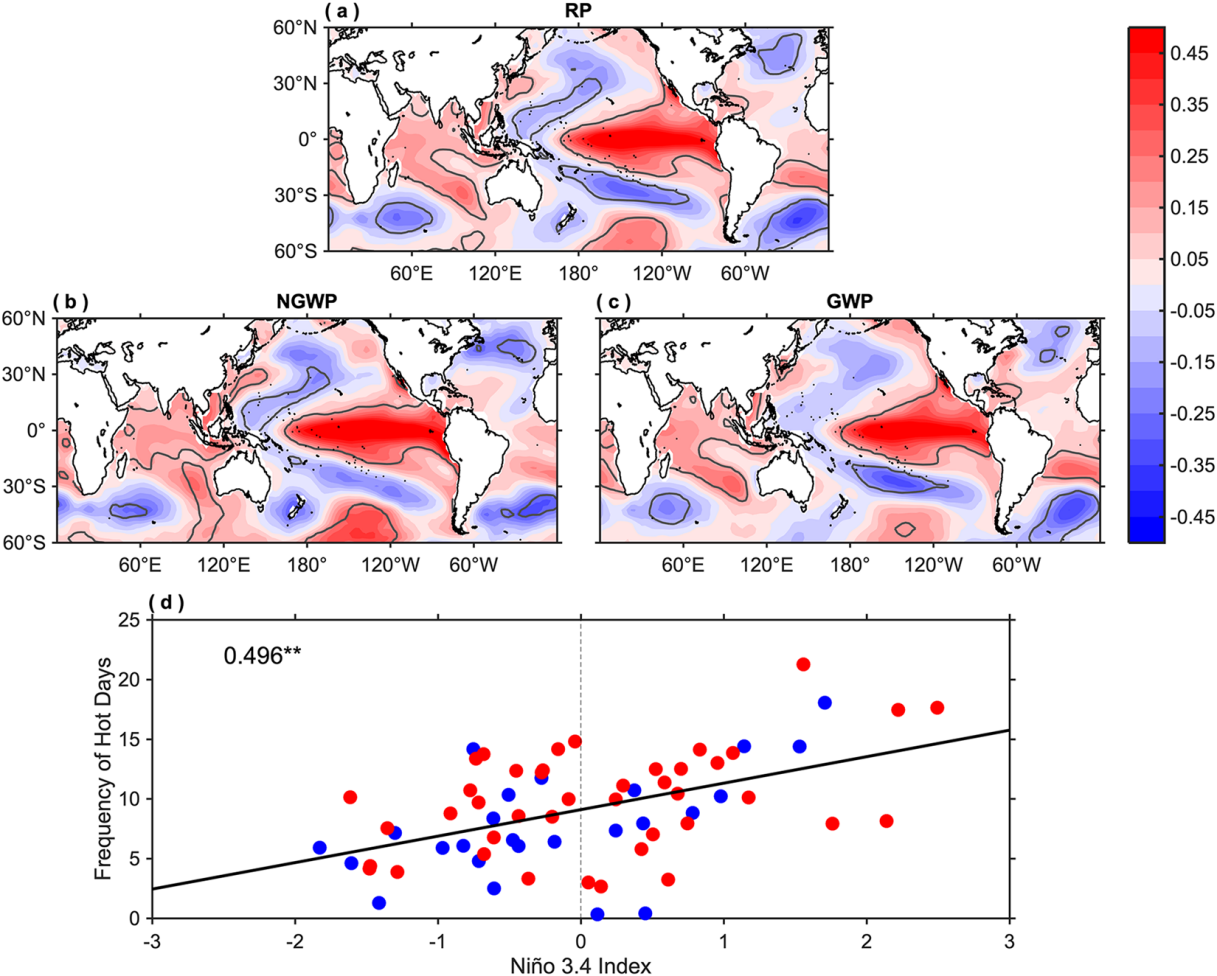
Role of ENSO in modulating hot extremes. (**a**–**c**) Regression of preceding DJF SSTAs (units are °C per standard deviation) onto the standardized de-trended frequency of hot days for the reference period (RP), non-global warming period (NGWP), and global warming period (GWP). The grey contours indicate the regions where the regression coefficient is statistically significant at the 95% confidence level. (**d**) Scatter plot of the frequency of hot days, area-averaged over all-India (shown in Fig. 2g) versus the preceding standardized DJF SSTAs, area-averaged over Niño-3.4 region (5°S–5°N, 170°W–120°W). **Indicates that correlation value is statistically significant at the 95% confidence level. The blue and red scatters represent the non-global and global warming years, respectively. This figure was prepared using the MATLAB version R2017a software (http://in.mathworks.com).

Besides atmospheric circulation and local parameters, low-frequency natural variability modes such as ENSO also influence temperature extremes over many regions of the globe^29–31^, including India^32^. To investigate further the sea surface temperature (SST) forcing of hot day frequency and also to separate the impact of global warming from natural variability, the preceding winter (December-February; DJF) SST anomalies (SSTAs) are regressed onto the standardized de-trended frequency of hot days (Fig. 7a–c). The results clearly indicate that ENSO is providing a substantial forcing for hot days in the Indian region during all periods considered. This is consistent with the previous study^30^, reporting that positive DJF Niño-3.4 SSTAs correspond to positive April surface air temperatures over Southeast Asia. The scatter plot between the frequency of hot days and DJF SSTAs area-averaged over Niño-3.4 region (5°S-5°N, 170°W-120°W) further confirms this relationship (Fig. 7d), as a strong and significant correlation (0.496) between the two is obtained. The mechanism for the lag of several months is likely related to the 3-4 months delayed response of the Indian Ocean SSTs to ENSO^47^. This is confirmed by the regression of the AMJ SSTAs onto the standardized frequency of hot days, which shows considerable warming of the Indian Ocean (Fig. S6a–c). To verify this further, the scatter plot between the frequency of hot days and AMJ SSTAs area-averaged over the equatorial Indian Ocean (EIO; 10°S-10°N, 50°E-100°E) is constructed, which divulges a strong and significant relationship (0.478) between the two (Fig. S6d). However, also the warming in the tropical Atlantic region following a warm ENSO event may contribute.

Further, to investigate the impact of global warming, the SSTAs are regressed onto the standardized trend of hot days frequency (Fig. S7). For reference and global warming periods, it is indeed found that a global warming pattern appears. The slight cooling in the eastern Pacific during the global warming period is a well-known feature in the recent warming trend and related to a strengthening of the Pacific Walker circulation^48–50^. As expected there is no indication that global warming has impacted the frequency of hot days in the non-global warming period. Therefore, the slow background warming in the reference and the global warming periods can overall explain the increasing trend in the frequency of hot days during these periods.

## Summary and Conclusion

Coming back to the concerns addressed at the end of the Introduction, it has been observed that (1) the probability distribution of daytime maximum temperatures has shifted towards higher values and has more spread, which insinuates more frequent hot days having higher temperatures in recent decades, compared to the past. The extreme temperatures upsurge in such a way that the 90^th^ percentile threshold of the non-global warming period equates to the 86^th^ percentile of the global warming period.

Regarding point (2), related with the effect of this shift on hot extremes (i.e., either on their frequency or intensity), it has been observed that there is a spatial shift in the grids illustrating significant increasing trends in the hot days frequency, i.e., during the non-global/global warming period, hot days frequency has significantly increased over southeast/western India. On the whole, the average frequency of hot days, area-averaged over all-India, has significantly increased by a factor of 24.7% in the recent climate, compared to the past. Furthermore, the climatological mean difference of hot extremes between the recent and the past climate reveals that the hot extremes have significantly increased across most parts of India (except over the Indo-Gangetic plains), indicating a clear shift in the climate towards warmer temperatures. The probable reason behind this is the intensive irrigation over the Indo-Gangetic plains that causes an increase in vegetation and evapotranspiration, which in turn induces a smaller portion of sensible heat flux versus latent heat over most parts of it.

**Figure 8:**
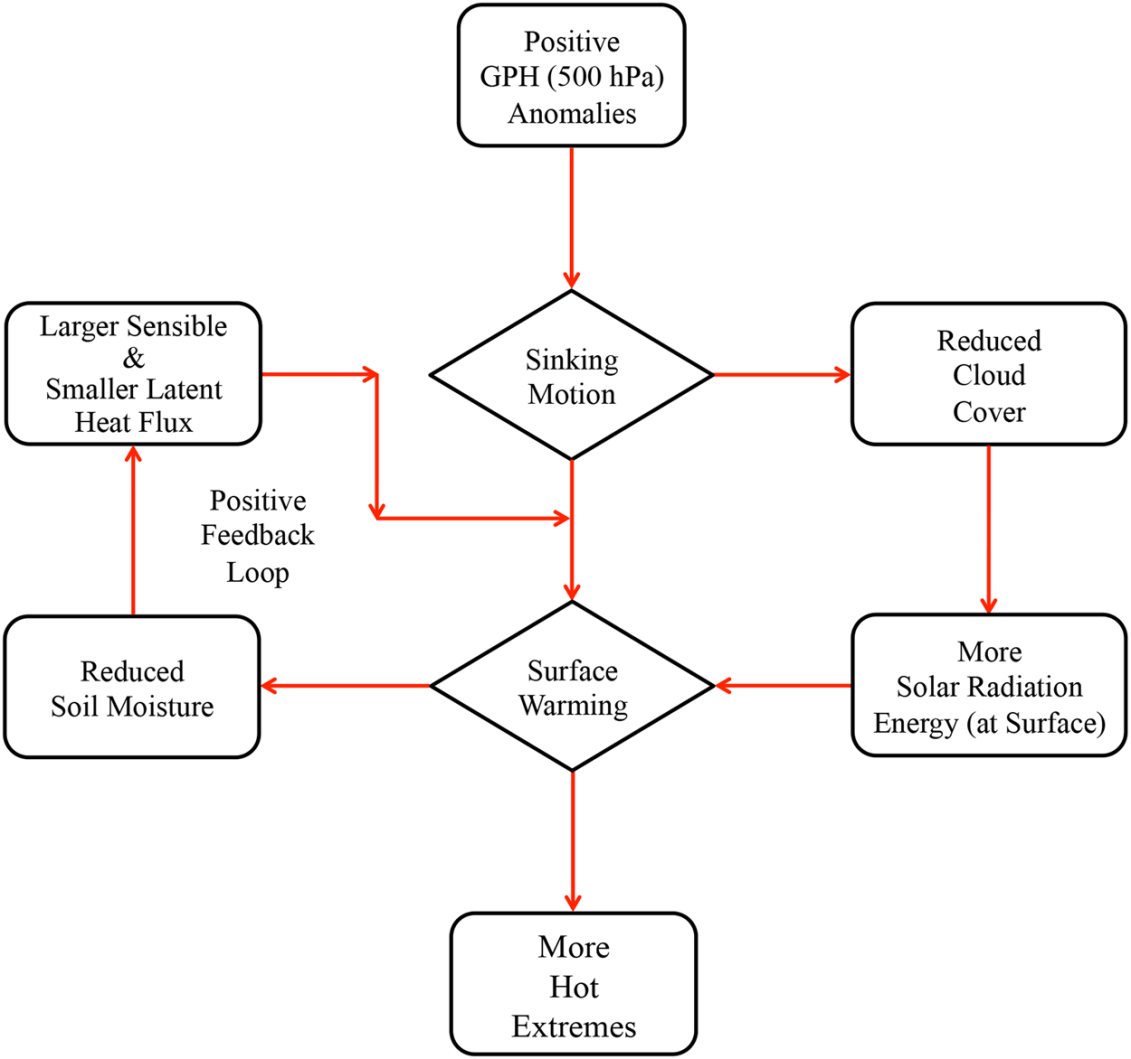
Schematic illustrating the physical mechanism associated with hot extremes.

Regarding point (3), the physical mechanism allied with these hot extremes, the key atmospheric circulation patterns, local parameters, and natural variability associated with ENSO are explored and conferred. In summary, Fig. 8 exemplifies a schematic of the physical mechanism linked with hot extremes. In the recent climate, the amplified geopotential height (abbreviated as GPH) anomalies at 500 hPa, compared to the past, is directly associated with hot extremes over India. This high-pressure system over the northern parts of India causes sinking motion that leads to surface warming due to adiabatic compression, which in turn causes more hot extremes. Furthermore, this sinking motion is associated with reduced cloud cover, which has allowed more solar radiation reaching the Earth’s surface that in turn leads to warmer temperatures over the Indian landmass. Additionally, the reduced soil moisture in the recent period has led to the larger proportion of sensible heat flux than latent heat flux into the atmosphere, inducing positive feedback between the atmospheric heating and further drying of the soil, which have resulted in an augmentation of near-surface air temperatures in the recent period. This study also investigates the role of natural variability like ENSO in modulating hot days over India. Results bestow that the preceding DJF SSTs over Niño-3.4 region provides substantial forcing for hot days (AMJ) over India during all periods considered and the lag of several months between Niño-3.4 SSTs and response in hot days is likely related to the 3–4 months delayed response of the Indian Ocean SSTs to ENSO.

The aspiration of this study is to enhance our knowledge about the processes governing the hot extremes over India. Furthermore, the results debated here have important implications for developing appropriate mitigation and adaptation strategies related to the consequences of extreme temperatures.

## Data and Methods

### Data

To characterize hot extremes over India, the high-resolution daily maximum temperature dataset for the period 1951–2018 from IMD is used in this study. The dataset was developed using the daily maximum temperature of 395 quality control stations all over India. The station data is interpolated to 1**°** × 1**°** latitude/longitude grids using the modified version of Shepard’s angular distance weighting algorithm. Errors were estimated and found less than 0.5 **°**C using root mean square errors. The errors are relatively larger in the hilly areas of Jammu and Kashmir and Uttarakhand due to the strong topographical gradient and data scarcity in those areas^51^.

To analyze the relationship of atmospheric and surface parameters with hot extremes, various variables such as geopotential height (500 hPa), vertical velocity (500 hPa), upward longwave radiation flux (top of the atmosphere), soil moisture (0–10 cm), precipitable water (for the entire atmosphere), relative humidity (850 hPa), downward solar radiation flux (surface), downward longwave radiation flux (surface), sensible, and latent heat fluxes at daily time scales are obtained from the National Center for Environmental Prediction (NCEP)/National Center for Atmospheric Research (NCAR) atmospheric reanalysis V1 for the period 1951–2018^52^. This dataset has been extensively used in several studies for exploring the atmospheric circulation features^15,18,27,28^.

To investigate the SST forcing of hot days frequency, the National Oceanic and Atmospheric Administration (NOAA) Extended Reconstructed SST version 5 (ERSST.v5)^53^ monthly data (resolution 2° x 2°) is obtained from http://www.ncdc.noaa.gov.

### Methods

To explore how the characteristics of hot extremes and allied mechanisms differ before and after the 1976 climate shift, the reference period (i.e., 1951–2018) is divided into the non-global (1951–1975) and global (1976–2018) warming periods as defined earlier^18,54,55^. To confirm that the maximum temperature over India also witnessed a climate shift in 1976, the change point (time instant at which some statistical property like mean, root-mean-square, standard deviation, or linearity of a signal changes abruptly) detection code available with MATLAB software is used^56,57^. Herein, the linear algorithm is applied to find the points where the slope and the mean change most abruptly. Using this algorithm on annual mean maximum temperature, area-averaged over all-India, two change points (i.e., 1964 and 1976) were detected. For the first two segments (i.e., 1951–1963 and 1964–1975), the trend is decreasing, while for the third one (i.e., 1976–2018), it is increasing. Thus, the year 1976 is considered to be a more influential change point because the maximum temperature decreased before that and increased afterwards.

The annual cycle of monthly mean maximum temperature, area-averaged over all-India, clearly reveals that the highest temperatures over India occur during the AMJ season (Fig. S8). That is why the AMJ season has been considered to explore the characteristics of hot extremes. The PDFs are computed using the daily maximum temperature anomalies for the AMJ season during 1951–1975 (sample size of 25 years × 91 days = 2275) and 1976–2018 (sample size of 43 years × 91 days = 3913). Relative frequencies are calculated based on the counts of maximum temperature anomalies between −6 **°**C and +6 **°**C, using a bin width of 0.1 **°**C. To estimate if the PDFs for the two time periods are significantly different from each other, a non-parametric two-sample Kolmogorov-Smirnov (K-S)^22,58^ test is applied. The two-sample K-S test evaluates the maximum absolute difference between the empirical cumulative distribution functions (CDFs) of two samples over the entire range in each dataset. The K-S test is defined as

1 $$D={\max }_{x}(|\widehat{{F}_{1}}(x)-\widehat{{F}_{2}}(x)|)$$

where $\widehat{{F}_{1}}(x)$ and $\widehat{{F}_{2}}(x)$ are the empirical CDFs of the non-global and global warming periods, respectively. The null hypothesis (H_0_) is that both samples come from the same distribution. If K-S = 1, then we will reject the null hypothesis at the 95% confidence level and accept the alternate hypothesis (H_a_: states that both samples come from different distributions). In this article, the entire reference period (i.e., 1951–2018) is used for computing the anomalies as well as the percentile thresholds. The purpose of selecting the complete reference period is to reduce the effects of multidecadal natural climate fluctuations^59^.

A percentile-based approach is used to estimate the extreme hot events based on the daily maximum temperatures for the AMJ season during the reference period. At each grid point, hot days are identified if the maximum temperature exceeds the 90^th^ percentile threshold. It is to be noted that the percentiles are computed only for those grids, which have at least 90% of data availability during the reference period. Out of the total 362 grids all over India, 28 grids in extreme north and northeast regions (shown by red; Fig. S9) have inconsistent data (i.e., less than 90% of data availability). Therefore, these grids are excluded from the analysis and set to as missing values. Finally, the number of days at each grid point having a maximum temperature above the 90^th^ percentile value is summed up for the AMJ season to see the spatial trend of its occurrence during respective periods.

Unlike the temperature data, the extreme temperature metrics usually do not follow a normal distribution as it represents the extreme states of the temperature. Therefore, the non-parametric statistics have been applied for identifying the magnitude and the significance of the trend. The advantage of using the non-parametric statistics is that it is distribution-free, i.e., it does not require data to be normally distributed. Besides this, the non-parametric statistics are also capable of handling the missing values in a dataset, which will likely occur due to the non-occurrence of a single hot extreme in a month/season/year.

For identifying the magnitude of trend, a non-parametric Sen’s slope estimator^60^ has been used. This method computes both the slope and the intercept according to Sen’s method. Firstly, the slope between each data pair (*x*_*i*_, *x*_*j*_) is computed using the following equation:

2 $${d}_{k}=\frac{{x}_{j}-{x}_{i}}{j-i}\,for\,(1\le i < j\le n)$$

where *d* is the slope, *x* denotes the variable, *n* is the number of data points in the time series, and *i* and j refer to the data of the indices. For n number of data points, one will get ${\rm{n}}\,({\rm{n}}-1)/2$ estimates of slope (d_k_). Sen’s slope is then defined as the median of all slopes, i.e., $b=Median\,{d}_{k}$. The intercepts are also computed for each time step (*t*) using the following equation, i.e., ${a}_{t}={x}_{t}-b\ast t$. Sen’s intercept is then defined as the median of all intercepts, i.e., $a=Median\,{a}_{t}$.

To detect the presence of a significant trend, non-parametric Mann-Kendall statistics^18,55,61–63^ is applied. Mann-Kendall statistics (*S*) is defined as the sum of the number of positive differences minus the number of negative differences, i.e.,

3 $$S=\mathop{\sum }\limits_{j=i+1}^{n}\mathop{\sum }\limits_{i=1}^{n-1}sign({x}_{j}-{x}_{i})$$

The positive value of *S* is an indicator of an increasing trend. Further, to assess the significance in the trend, the normalized test statistic (Z) and the statistical probability or confidence in trend ($1-{\rm{p}}$) is computed; where *p* is the probability density function for normal distribution and is defined as

4 $${\rm{p}}={\rm{f}}({\rm{Z}})=\frac{1}{\sqrt{2{\rm{\pi }}\,}}{{\rm{e}}}^{-\frac{{{\rm{Z}}}^{2}}{2}}$$

But before calculating this, the variance of S is computed by using the following equation, i.e.,

5 $${\rm{Var}}({\rm{S}})=\frac{1}{18}\{{\rm{n}}({\rm{n}}-1)(2{\rm{n}}+5)-\mathop{\sum }\limits_{{\rm{p}}=1}^{{\rm{g}}}{{\rm{t}}}_{{\rm{p}}}({{\rm{t}}}_{{\rm{p}}}-1)(2{{\rm{t}}}_{{\rm{p}}}+5)\}$$

where *n* is the number of data points, *g* is the number of tied groups, and t_p_ is the number of data points in the p^th^ group. Now the normalized test statistic, *Z*, is defined as

6 $${\rm{Z}}=\{\begin{array}{ll}\frac{({\rm{S}}-1)}{\sqrt{{\rm{Var}}({\rm{S}})}} & if\,S > 0\\ 0 & if\,S=0\\ \frac{({\rm{S}}+1)}{\sqrt{{\rm{Var}}({\rm{S}})}} & if\,S < 0\,\end{array}$$

hence the trend is said to be increasing (decreasing) if Z is positive (negative) and the statistical probability is greater than the level of significance (i.e., at the 95% confidence level). Herein, the increasing/decreasing tendency refers to the statistically non-significant increasing/decreasing trends.

The statistical significance of the climatological mean difference shown in Fig. 4 is evaluated using the Student’s t-test for unequal variances^64,65^; whereas, the statistical significance of composites shown in Figs. 5, S4, and S5 is computed using Student’s one sample t-test. The statistical significance of regression coefficients shown in regression maps is assessed via a two-tailed t-test^66–68^.

To see, how do the atmospheric circulation and local parameters differ in the two periods, composite analysis is used; whereas to scrutinize the SST forcing of hot day frequency and to understand the impact of global warming on the hot extremes, regression analysis is applied. The composites of variables are constructed for the days when the area-averaged maximum temperature over India exceeds the 90^th^ percentile threshold for the reference period. To explore the extent of the relationship of atmospheric and surface parameters as well as natural variability with hot extremes, scatter plots are considered.

In this study, the geopotential height and vertical velocity are considered at 500 hPa because it is the level of maximum vertical motion and is generally referred to as the level of non-divergence, so it is an appropriate indicator for vertical movements. On the other hand, the rationale for considering relative humidity at 850 hPa is that its high values at this pressure level indicate the availability of moisture. Since the resolution of the variables from the NCEP/NCAR reanalysis dataset and the SST data differs, therefore, for ease of comparison the variables are interpolated into a common latitude-longitude grid (2.5° × 2.5°) by bilinear interpolation.

## Supplementary Information

Supplementary Information.
