# Supplementary material for: Assessing Changes in Characteristics of Hot Extremes Over India in a Warming Environment and their Driving Mechanisms
Source: Sci Rep. 2020 Feb 14;10:2631. doi: 10.1038/s41598-020-59427-z (PMC7021811; doi:10.1038/s41598-020-59427-z)
Supplement: Supplementary file 1 — Supplementary Information. [file 41598_2020_59427_MOESM1_ESM.pdf]

Supplementary Material

ASSESSING CHANGES IN CHARACTERISTICS OF HOT EXTREMES OVER INDIA IN A WARMING ENVIRONMENT AND THEIR DRIVING MECHANISMS

MANISH K. JOSHI [1,*]

[1] Indian Institute of Tropical Meteorology, Pune-411008, Maharashtra, India

ARCHANA RAI [1]

[1] Indian Institute of Tropical Meteorology, Pune-411008, Maharashtra, India

ASHWINI KULKARNI [1]

[1] Indian Institute of Tropical Meteorology, Pune-411008, Maharashtra, India

And

FRED KUCHARSKI [2,3]

[2] Earth System Physics Section, Abdus Salam International Centre for Theoretical Physics, Trieste, Italy

[3] Center of Excellence for Climate Change Research/Department of Meteorology, King Abdulaziz University, Jeddah, Saudi Arabia

* *Corresponding author's e-mail address:* manishkumarjoshi@gmail.com; dr.manishkjoshi@gmail.com; and manishkjoshi@tropmet.res.in

ORCID ID: - https://orcid.org/0000-0001-6498-6962

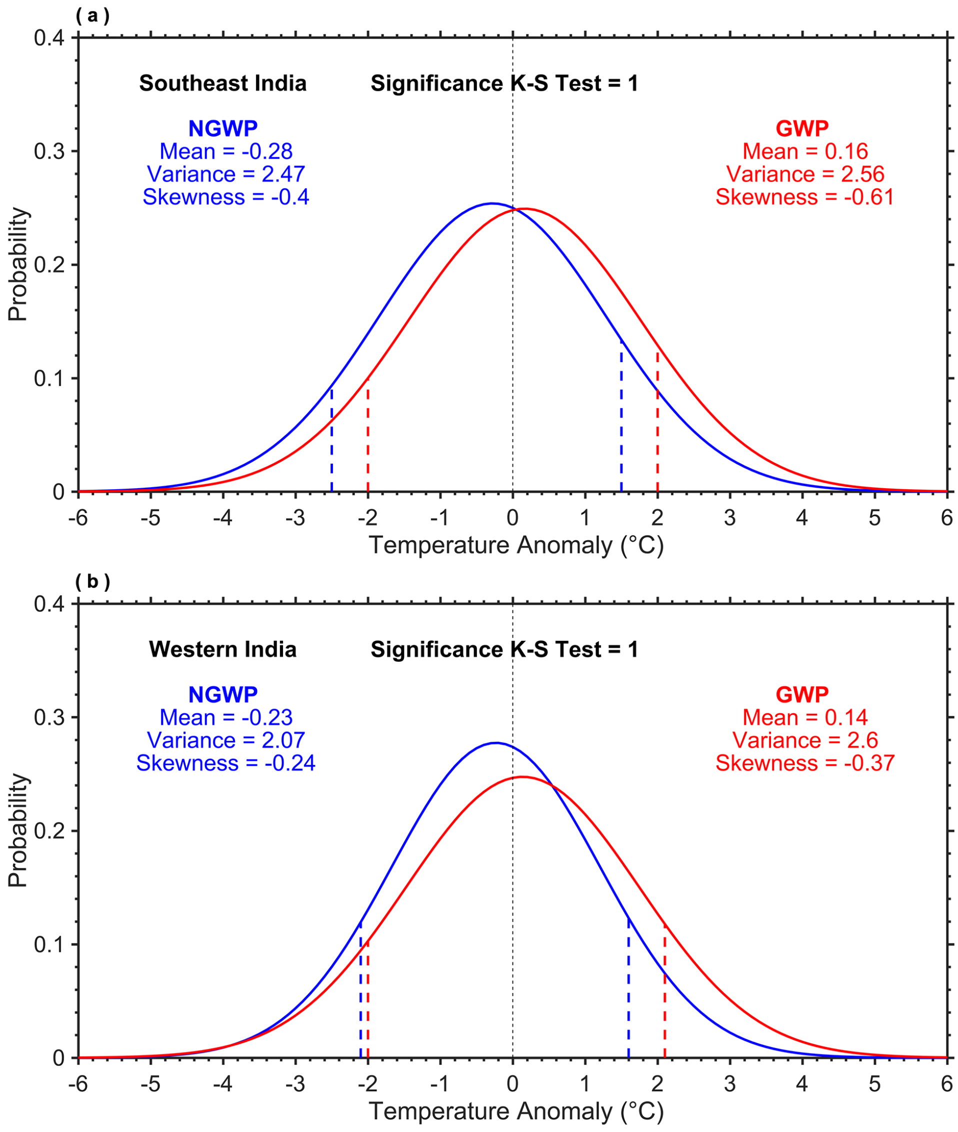

Figure S1. Shift in the probability distribution of daytime maximum temperatures for southeast and western India. Probability density functions of daily maximum temperature anomalies during the non-global warming period (NGWP: 1951-1975; blue) and global warming period (GWP: 1976-2018; red) for the AMJ season over (**a**) southeast (9.5°N–21.5°N, 76.5°E–84.5°E; area enclosed within the box shown in Fig. 2b) and (**b**) western (15.5°N–28.5°N, 68.5°E–76.5°E; area enclosed within the box shown in Fig. 2c) India. The K-S=1 reveals that the probability density functions for the two periods are statistically different from each other at 95% confidence level. The vertical lines represent the 10th and 90th percentiles of the respective distribution.

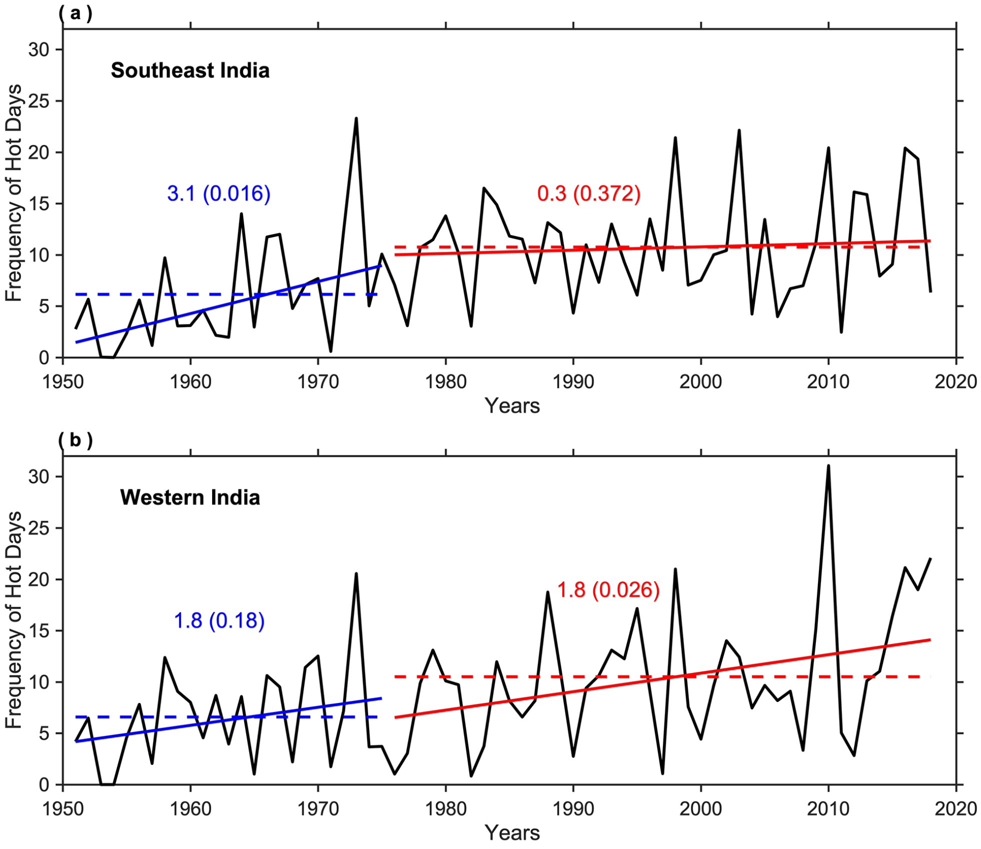

Figure S2. Temporal variation in the frequency of hot days for southeast and western India. Variation (black) and linear trends in the frequency of hot days, area-averaged over (**a**) southeast (9.5°N–21.5°N, 76.5°E–84.5°E; area enclosed within the box shown in Fig. 2b) and (**b**) western (15.5°N–28.5°N, 68.5°E–76.5°E; area enclosed within the box shown in Fig. 2c) India, during the non-global (blue) and global (red) warming periods for the AMJ season. Values along the trend lines indicate the amount of change in the number of hot days per decade and those in parentheses represent its p-values. The dashed lines indicate the average frequency of hot days in the non-global (blue) and global (red) warming periods.

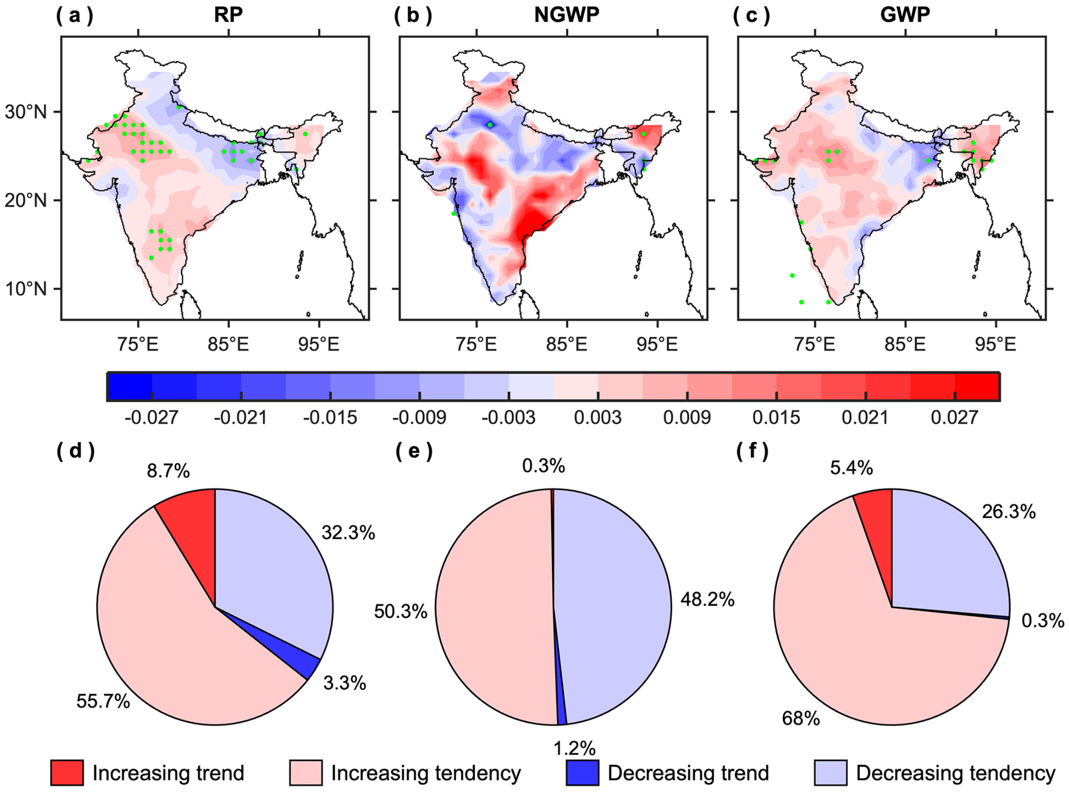

Figure S3. Trends in the average intensity of hot days. (a-c) Spatial patterns of temporal trends (°C/yr) and **(d-f)** corresponding pie charts, illustrating the number of grids (in percent) having increasing and decreasing trend/tendency, in the average intensity of hot days for the AMJ season during the reference period (RP: 1951-2018), non-global warming period (NGWP: 1951-1975), and global warming period (GWP: 1976-2018). The green stippling indicates the grid points where the trends are statistically significant at the 95% confidence level. This figure was prepared using the MATLAB version R2017a software (http://in.mathworks.com).

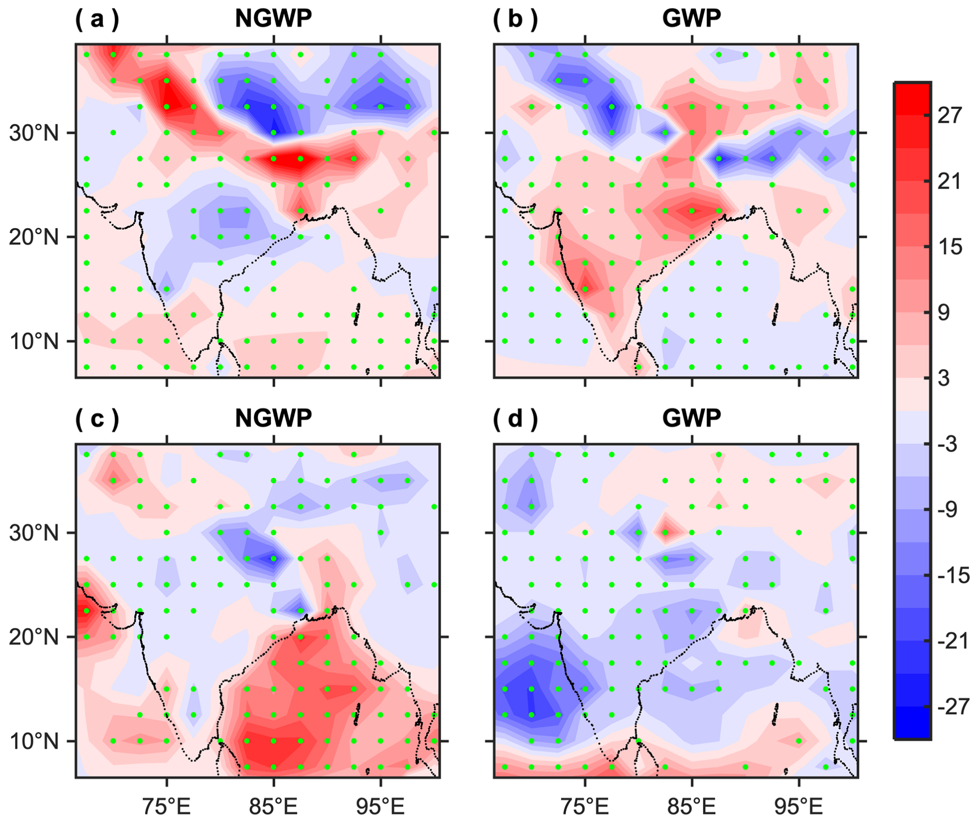

Figure S4. Composites of heat fluxes associated with hot extremes. Composites of (**a,b**) sensible heat flux (W/m^2) anomalies and (**c,d**) latent heat flux (W/m^2) anomalies corresponding to hot days for the AMJ season during the non-global warming period (NGWP: 1951-1975) and global warming period (GWP: 1976-2018), respectively. The green stippling indicates the grid points where the composite is statistically significant at the 95% confidence level. This figure was prepared using the MATLAB version R2017a software (http://in.mathworks.com).

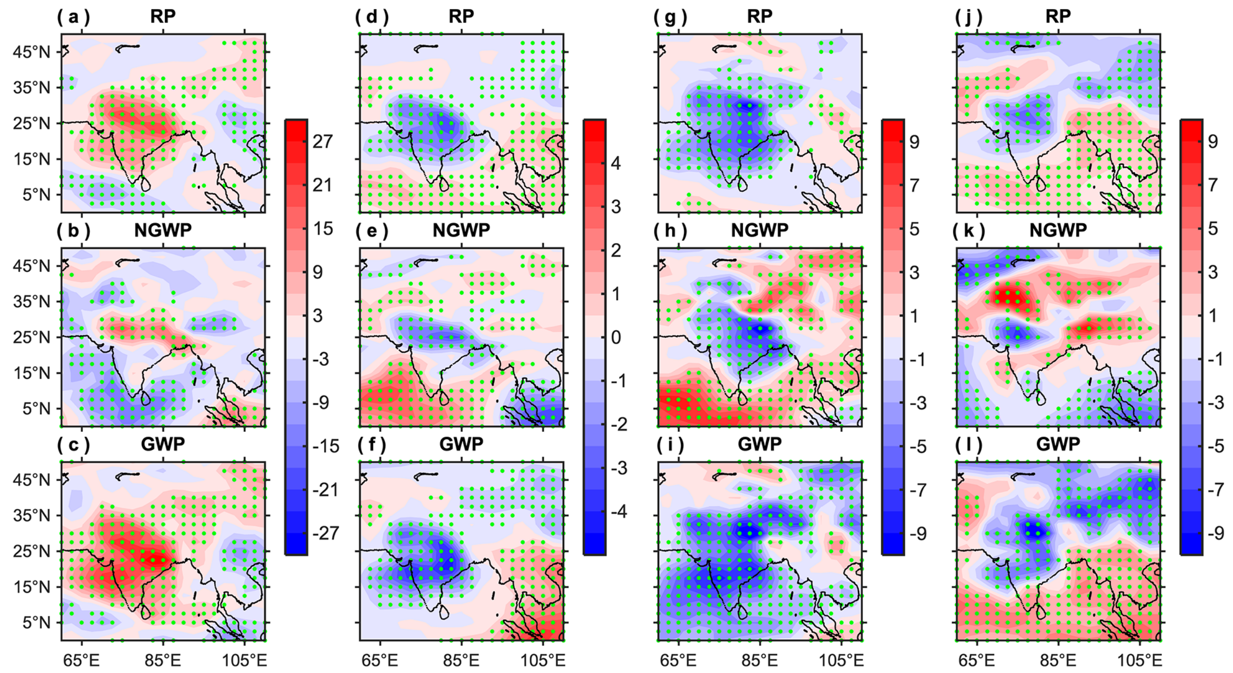

Figure S5. Composites of atmospheric parameters associated with hot extremes. Composites of (**a-c**) downward solar radiation flux (W/m^2) anomalies at surface, (**d-f**) precipitable water (kg/m^2) anomalies for entire atmosphere, (**g-i**) relative humidity (%) anomalies at 850 hPa, and (**j-l**) downward longwave radiation flux (W/m^2) anomalies at surface corresponding to hot days for the AMJ season during the reference period (RP: 1951-2018), non-global warming period (NGWP: 1951-1975), and global warming period (GWP: 1976-2018), respectively. The green stippling indicates the grid points where the composite is statistically significant at the 95% confidence level. This figure was prepared using the MATLAB version R2017a software (http://in.mathworks.com).

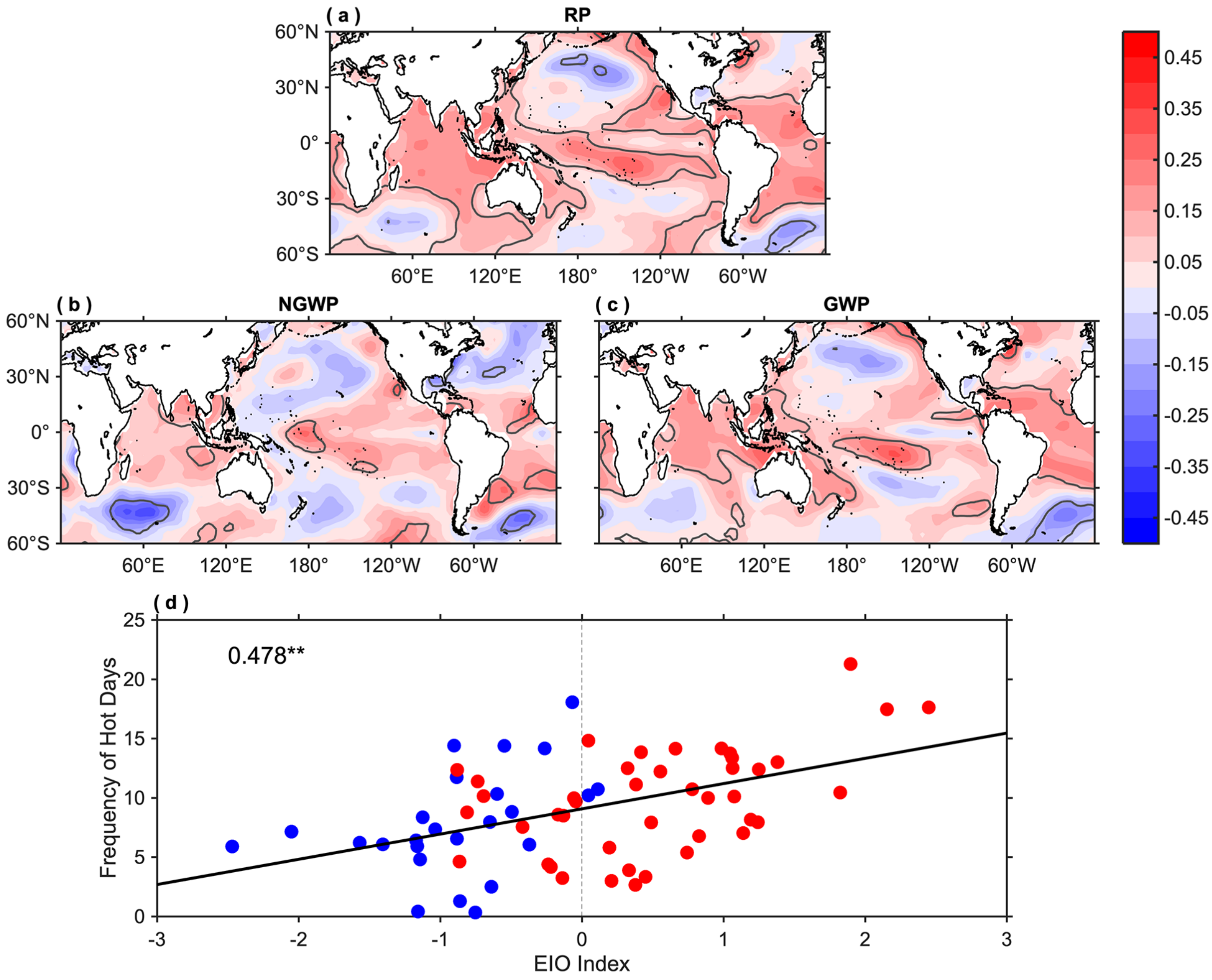

Figure S6. Role of Indian Ocean SSTs in modulating hot extremes. (**a-c**) Regression of AMJ SSTAs (units are °C per standard deviation) onto the standardized frequency of hot days for the reference period (RP), non-global warming period (NGWP), and global warming period (GWP). The grey contours indicate the regions where the regression coefficient is statistically significant at 95% confidence level. (**d**) Scatter plot of the frequency of hot days, area-averaged over all-India versus the standardized AMJ SSTAs, area-averaged over the equatorial Indian Ocean region (EIO; 10°S–10°N, 50°E–100°E). ** Indicates that correlation value is statistically significant at the 95% confidence level. The blue and red scatters represent the non-global and global warming years, respectively. This figure was prepared using the MATLAB version R2017a software (http://in.mathworks.com).

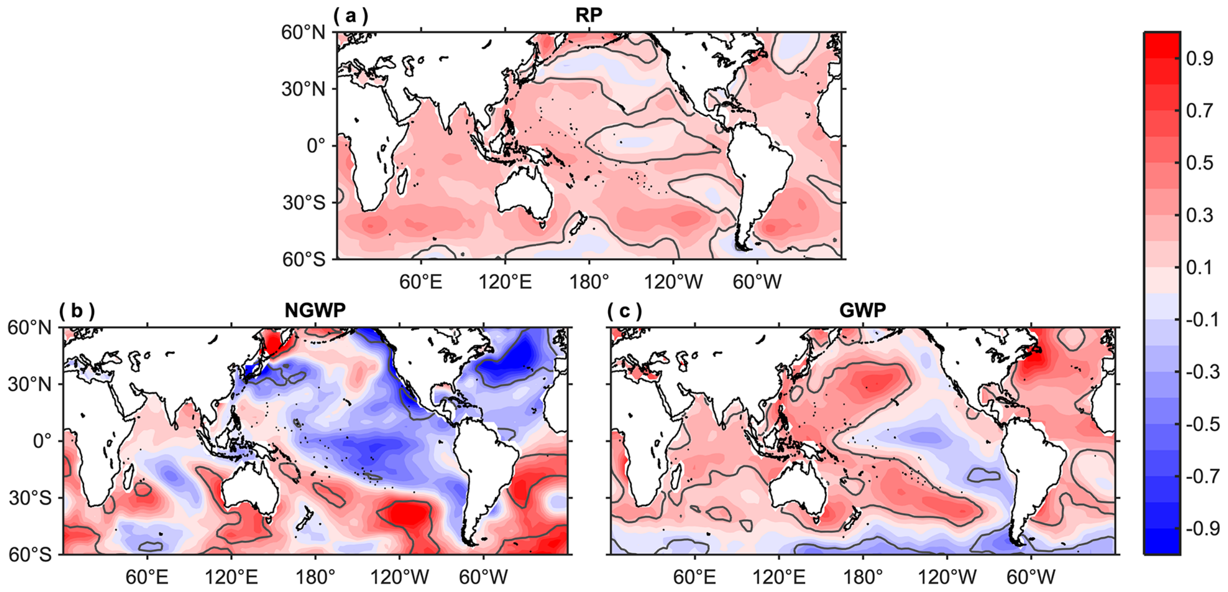

Figure S7. Impact of Global Warming on hot extremes. (**a-c**) Regression of preceding DJF SSTAs (units are °C per standard deviation) onto the standardized trend of hot days frequency for the reference period (RP), non-global warming period (NGWP), and global warming period (GWP). The grey contours indicate the regions where the regression coefficient is statistically significant at the 95% confidence level. This figure was prepared using the MATLAB version R2017a software (http://in.mathworks.com).

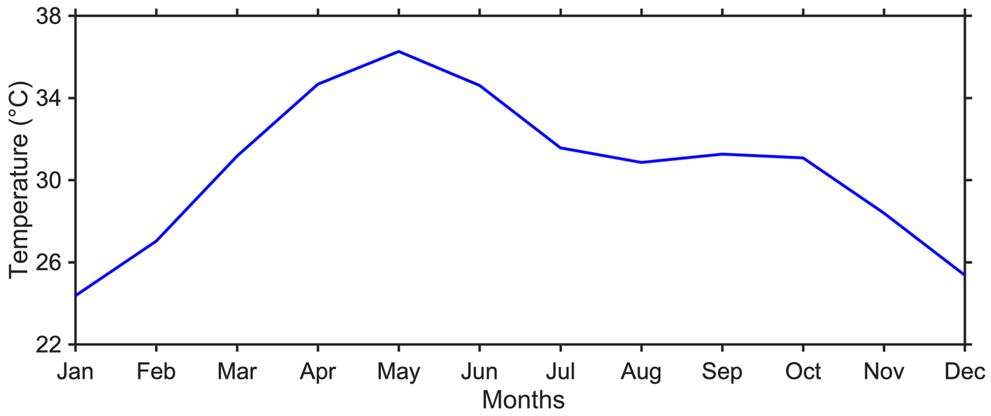

Figure S8. Annual cycle of monthly mean maximum temperature (°C), area-averaged over all-India.

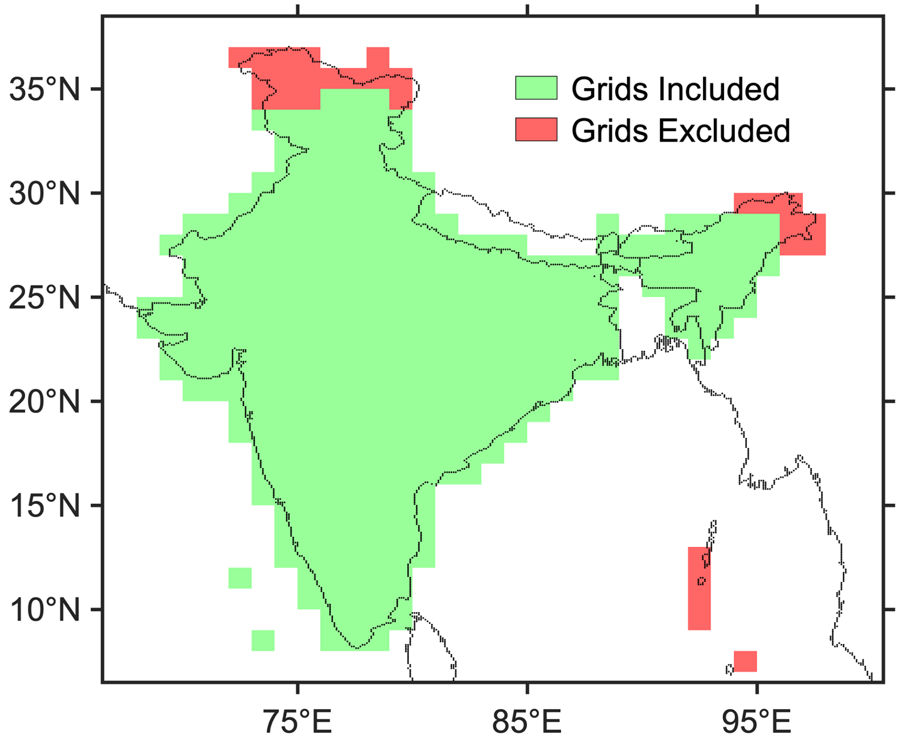

Figure S9. Map illustrating the grid points of IMD maximum temperature data, which are included (having at least 90% of data availability during the reference period) and excluded (having less than 90% of data availability during the reference period) in this study. This figure was prepared using the MATLAB version R2017a software (http://in.mathworks.com).

Table S1. Spatial consistency between the frequency of hot days and their corresponding average intensity using cross-tabulation and chi-square statistics for the reference period (1951-2018; values in black), non-global warming period (1951-1975; values in blue), and global warming period (1976-2018; values in red). *

		Average Intensity of Hot Days			
		Increasing Trend	Increasing Tendency	Decreasing Trend	Decreasing Tendency
Frequency of Hot Days	Increasing Trend	**26** / **1** / **13**	**139** / **44** / **36**	**0** / **0** / **0**	**10** / **16** / **5**
	Increasing Tendency	**3** / **0** / **5**	**45** / **123** / **182**	**1** / **2** / **0**	**37** / **138** / **60**
	Decreasing Trend	**0** / **0** / **0**	**0** / **0** / **0**	**5** / **0** / **1**	**9** / **0** / **0**
	Decreasing Tendency	**0** / **0** / **0**	**2** / **1** / **9**	**5** / **2** / **0**	**52** / **7** / **23**

* The chi-square statistics for the reference, non-global, and global warming periods are 230.42 (0.00), 52.90 (0.00), and 416.12 (0.00), respectively. The values in parentheses represent their respective p-values.

Table S2. Spatial consistency between the frequency of hot days and seasonal maximum intensity using cross-tabulation and chi-square statistics for the reference period (1951-2018; values in black), non-global warming period (1951-1975; values in blue), and global warming period (1976-2018; values in red). *

		Seasonal Maximum Intensity			
		Increasing Trend	Increasing Tendency	Decreasing Trend	Decreasing Tendency
Frequency of Hot Days	Increasing Trend	**145** / **40** / **31**	**30** / **21** / **23**	**0** / **0** / **0**	**0** / **0** / **0**
	Increasing Tendency	**10** / **6** / **35**	**57** / **211** / **171**	**1** / **0** / **0**	**18** / **46** / **41**
	Decreasing Trend	**0** / **0** / **0**	**0** / **0** / **0**	**11** / **0** / **1**	**3** / **0** / **0**
	Decreasing Tendency	**0** / **0** / **0**	**5** / **2** / **7**	**3** / **1** / **4**	**51** / **7** / **21**

* The chi-square statistics for the reference, non-global, and global warming periods are 505.82 (0.00), 224.94 (0.00), and 207.80 (0.00), respectively. The values in parentheses represent their respective p-values.

Table S3. Spatial consistency between the non-global (1951-1975) and global (1976-2018) warming periods for the frequency of hot days (values in black), average intensity of hot days (values in blue), and maximum intensity (values in red) using cross-tabulation and chi-square statistics. *

<table>
<tr><td colspan="2" rowspan="2"></td><td colspan="4">Global Warming Period</td></tr>
<tr><td>Increasing Trend</td><td>Increasing Tendency</td><td>Decreasing Trend</td><td>Decreasing Tendency</td></tr>
<tr><td rowspan="4">Non-Global Warming Period</td><td>Increasing Trend</td><td>5 / 0 / 0</td><td>48 / 1 / 25</td><td>0 / 0 / 0</td><td>8 / 0 / 21</td></tr>
<tr><td>Increasing Tendency</td><td>46 / 6 / 54</td><td>193 / 112 / 144</td><td>1 / 1 / 5</td><td>23 / 49 / 31</td></tr>
<tr><td>Decreasing Trend</td><td>0 / 2 / 1</td><td>0 / 2 / 0</td><td>0 / 0 / 0</td><td>0 / 0 / 0</td></tr>
<tr><td>Decreasing Tendency</td><td>3 / 10 / 11</td><td>6 / 112 / 32</td><td>0 / 0 / 0</td><td>1 / 39 / 10</td></tr>
</table>

* The chi-square statistics for frequency of hot days, average intensity of hot days, and maximum intensity are 5.58 (0.4714), 19.59 (0.0206), and 38.68 (0.00), respectively. The values in parentheses represent their respective p-values.
